# Overall survival after recurrence in stage I–III colorectal cancer patients in accordance with the recurrence organ site and pattern

**DOI:** 10.1002/ags3.12483

**Published:** 2021-07-14

**Authors:** Hiroshi Sawayama, Yuji Miyamoto, Yukiharu Hiyoshi, Katsuhiro Ogawa, Rikako Kato, Takahiko Akiyama, Yuki Kiyozumi, Naoya Yoshida, Hideo Baba

**Affiliations:** ^1^ Department of Gastroenterological Surgery Graduate School of Medical Sciences Kumamoto University Kumamoto Japan; ^2^ Department of Gastroenterological Surgery Cancer Institute Hospital of Japanese Foundation for Cancer Research Tokyo Japan

**Keywords:** colorectal cancer, oligometastasis, prognosis, recurrence organ, recurrence pattern

## Abstract

**Aim:**

This study aimed to investigate the prognosis after recurrence in patients with stage I–III colon cancer (CC) and rectal cancer (RC).

**Methods:**

Cancer recurred in 116 (15.2%) out of 763 patients with stage I–III colorectal cancer. The overall survival (OS) after recurrence was evaluated based on the recurrence organs and patterns.

**Results:**

The first recurrence occurred in the lungs, livers, lymph nodes, and other sites in 32, 22, 12, and 2 patients, respectively. It was localized, disseminated, and involved two or more organs in 14, 9, and 25 patients, respectively. Patients with CC had a shorter OS after recurrence as compared to those with RC (*P* = .0103). Compared to other organ metastasis, liver metastasis was associated with an earlier recurrence (*P* = .0026) and shorter OS after recurrence (hazard ratio [HR]: 2.216; 95% confidence interval [CI]: 1.052–4.459; *P* = .0370). Lung metastasis was associated with a more favorable prognosis as compared to other organ recurrences (HR: 0.338; 95% CI: 0.135–0.741; *P* = .0057). One‐organ recurrence and oligometastasis were observed in 78.4% and 49.1% of the patients, respectively. The 5‐y OS rates of patients with one‐organ recurrence and oligometastasis were 47.5% and 71.7%, respectively. Invasive treatment was associated with a favorable prognosis (*P* < .0001).

**Conclusions:**

Liver metastasis and dissemination were associated with a shorter OS after recurrence. Approximately 50% of the patients experienced oligometastasis, which was associated with a favorable prognosis. Hence, to improve patient prognosis it is better to perform invasive treatments when possible.

## Graphical abstract

One organ recurrence was observed in 78.4% of patients and oligometastasis in 49.1%. The 5‐y OS rates of patients with one organ recurrence and oligometastasis were 47.5% and 71.7%, respectively. Invasive treatment was associated with a favorable prognosis (*P* < .0001).

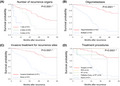

## 1 INTRODUCTION

Colorectal cancer (CRC) is the second and third most commonly diagnosed malignancy in women and men worldwide, respectively.^1^ Recurrence has been observed in 18.7% of the patients with stage I–III CRC; the recurrence organ was reportedly associated with the primary lesion in accordance with the Japanese Society for Cancer of the Colon and Rectum guidelines. Patients with colon cancer (CC) reportedly experienced peritoneal metastasis more frequently as compared to those with rectal cancer (RC). Conversely, patients with RC reportedly experienced lung metastasis and local recurrence more frequently as compared to those with CC. Patients with stage II–III CRC experienced an earlier recurrence as compared to those with stage I CRC. The cumulative incidence of recurrence, accordance to the recurrence site, was demonstrated in a previous study^2^; however, there was no evidence of an association between the recurrence organ site and pattern and the overall survival (OS) after recurrence.^3^

In a previous study, the OS of patients with stage IV CRC was difficult to estimate based on the metastatic lesions, because the degree of progression of CRC differed in each patient. Various triggers were diagnosed as CRC with distant metastasis, such as stenosis of the primary lesion, bleeding, and detection of fecal occult blood. The metastatic organ sites and number of metastases are known to vary, and the treatment strategy for metastatic lesions differs according to the status of distant metastasis. In our study, patients with stage I–III CRC who underwent surgery were followed up in accordance with the treatment guidelines.^2^ Recurrence was diagnosed during the early phase of widespread metastasis. The metastatic organ site and the number of metastases can be estimated with precision using diagnostic imaging.

The concept of oligometastasis was reported by Hellman and Weichselbaum.^4^ Sentinel lymph nodes (LN) may be the first site of recurrence from the primary lesion. Metastatic lesions are initially detected in a limited area of a distant organ; they then metastasize to involve multiple organs. Oligometastasis is an intermediate state between localized disease and widespread metastases. There have been a few reports on oligometastasis in CRC.^5^ The aim of this retrospective study was to investigate the prognosis of CRC patients with recurrence, in terms of the recurrence organs, recurrence patterns, number of recurrence organs, and treatment for the recurrence sites.

## 2 MATERIALS AND METHODS

### 2.1 Patients

In this study we included 763 patients with stage I–III CRC who underwent primary resection at the Kumamoto University Hospital between April 2005 and March 2019. These patients were analyzed for the recurrence organ, recurrence pattern, and number of recurrences. The clinical and pathological characteristics of the patients were retrieved from the medical record database to analyze the association between the prognosis and recurrence organs and patterns. Recurrence occurred in 116 of the 763 patients. Hence, 116 patients were available for the analysis of first organ recurrence and survival after recurrence. In this study surgical resection (n = 44), radiofrequency ablation (n = 2), and radiation therapy (n = 5) were considered invasive treatments, while palliative surgery and palliative radiation therapy (n = 6) were not. Therefore, 51 patients underwent invasive treatments. Of the remaining 65 patients, 29 underwent chemotherapy after recurrence in our hospital, 11 underwent neither chemotherapy nor invasive treatment, and 25 were not followed up for chemotherapy in detail. Further, we defined both one‐organ recurrence and the presence of fewer than five lesions in the organ as oligometastasis, in accordance with previous studies.^6^ Data on the number of lesions in a recurrent organ and on invasive treatment were unavailable for 10 patients with one‐organ recurrence and 8 patients, respectively. Hence, these patients were excluded from the analysis of oligometastasis and invasive treatment. Written informed consent was obtained from all patients.

### 2.2 Clinicopathological characteristics of the patients

We observed that right‐sided CC was proximal to the splenic flexure and left‐sided CC was distal to the splenic flexure, without the involvement of the rectum; this was in accordance with recent reports.^7^ Tumor location was classified in accordance with the Japanese classification of colorectal, appendiceal, and anal carcinoma.^8^ Therefore, the rectal sigmoid (defined as the segment from the height of the sacral promontory to the inferior border of the second sacral vertebra) was considered the rectum. Clinical data, including age, sex, body mass index, TNM stage, preoperative carcinoembryonic antigen titer, and preoperative carbohydrate antigen 19‐9 titer, were retrospectively available for 116 patients. The cutoff value for each was based on the recommendations of the measuring kits adopted by our institute.

### 2.3 Treatment strategy and follow‐up evaluation

The treatment strategy and follow‐up evaluation were in accordance with the Japanese colorectal cancer guidelines. Primary resection with LN dissection was recommended for Stage I–III CRC. Of the 116 patients, 3 (2.6%), 17 (14.6%), and 96 (82.8%) patients underwent D1, D2, and D3 LN dissection, respectively. After the surgery, the patients were followed up at 3‐mo intervals. Recurrence was confirmed by clinical examinations including computed tomography (CT). Tumor marker levels were measured every 3 mo, and CT scanning studies from the neck to the pelvis were performed at least twice a year for 5 y after the surgery.

### 2.4 Adjuvant chemotherapy

For adjuvant chemotherapy, stage III and high‐risk stage II patients received monotherapy (UFT/UZEL, Capecitabine or S‐1) or doublet therapy (CAPOX or SOX regimen) for 3–6 mo. None of the 10 stage I patients received adjuvant chemotherapy. Of the 40 stage II patients, four (10%) and one (2.5%) patients received monotherapy and doublet therapy, respectively. Furthermore, of the 66 stage III patients, 20 (30.3%) and 19 (28.8%) patients received monotherapy and doublet therapy, respectively. In the case of eight patients, it was unclear whether they received adjuvant chemotherapy or not.

### 2.5 Statistical analyses

The relationship between primary lesions and the clinicopathological characteristics of the patients was determined using the chi‐squared test. Mortality was estimated using the recurrence rate and the OS after recurrence. The Wilcoxon signed‐rank test was used to evaluate early recurrence. The log‐rank test and univariate Cox proportional hazard regression analysis were used to analyze the OS, and the Kaplan–Meier estimator was used to assess the distribution of survival time. All *P* values were two‐sided, and statistical significance was set at *P* < .05. The results are presented as hazard ratios (HRs) and 95% confidence intervals (CIs). All data were processed and analyzed using the JMP 11 software (SAS Institute, Cary, NC).

## 3 RESULTS

### 3.1 Incidence of recurrence and overall survival

A total of 763 patients underwent surgery for stage I–III CRC. Over a median follow‐up of 36.4 mo, 116 (15.2%) cases experienced recurrence. The incidence of recurrence varied according to the site of the primary lesion. Recurrence was detected in 11.0% (26/236), 13.3% (33/248), and 20.4% (57/279) of the patients with right‐sided primary lesions, left‐sided primary lesions, and primary lesions in the rectum, respectively. The recurrence rates in stage I, stage II, and stage III patients were 3.7% (10/267), 14.9% (40/268), and 28.9% (66/228), respectively. The 5‐y OS rate in all 763 patients was 81.9%.

### 3.2 Association between tumor location and prognosis

A total of 488 patients (64.0%) had CC (right‐sided: 247 [32.4%], left‐sided: 241 [31.6%]), while 275 patients (36.0%) had RC. Univariate analysis revealed that RC was significantly associated with a younger age (*P* < .0001), advanced tumor stage (*P* = .0031), and the presence of LN metastasis (*P* = .0007) (Table S1). The 5‐y recurrence rate was higher in patients with RC (23.3%) than in patients with CC (14.1%) (*P* = .0018; Figure S1A and Table S2). However, the 5‐y OS tended to be longer in patients with RC (84.3%) than in those with CC (80.6%) (*P* = .0652; Figure S1B and Table S3). Multivariate analysis also revealed that the recurrence rate was higher in patients with RC than in those with CC (Table S2); however, no significant differences in the OS rate were noted between patients with RC and CC (Table S3).

### 3.3 Recurrence organs and survival according to the primary tumor location

**FIGURE 1:**
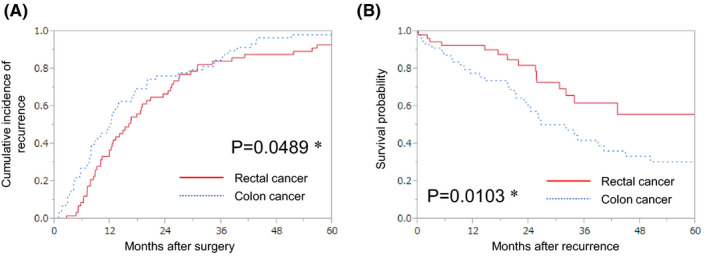
Recurrence rate and survival after recurrence according to the primary lesion. Kaplan–Meier survival curve for the recurrence rate (*P* value was estimated using the Wilcoxon signed‐rank test) (A). Kaplan–Meier curve for the overall survival after recurrence (*P* value was estimated using the log‐rank test) (B). *Statistically significant

The 116 patients with recurrence were evaluated on the basis of the primary tumor location and recurrence organ sites. Patients with recurrent RC were significantly younger than those with recurrent right‐ or left‐sided CC (*P* = .0245). Other clinicopathological characteristics were not associated with the primary lesion location (Table 1). Patients with right‐ or left‐sided CC had an earlier recurrence (Wilcoxon test, *P* = .0489) and a shorter OS after recurrence (*P* = .0103) as compared to those with RC (Figure 1). The first recurrence occurred at one site in 91 cases (78.4%), two sites in 20 cases (17.2%), three sites in three cases (2.6%), and four sites in two cases (1.7%) (Table 2). The recurrence organ site was more frequently localized in RC (*P* = .0408) as compared to in right‐ or left‐sided CC, and tended to accompany lung recurrence when the patients had two or more organ recurrences (*P* = .0618). The liver was the more frequent recurrence organ site in right‐ or left‐sided CC (*P* = .0313) as compared to in RC, and was accompanied by dissemination when the patients had two or more organ recurrences (*P* = .0073; Table 3 and Table S4).

**TABLE 1:** Association between the primary lesion and clinicopathological factors

Factors	Total N	Colon N = 59	[R/L]		Rectum N = 57		*P*‐value
Age							
≤70	61	25	[13/12]	(42.4%)	36	(63.2%)	.0245^a^
>70	55	34	[22/12]	(57.6%)	21	(36.8%)	
Gender							
Male	56	27	[13/14]	(45.8%)	29	(50.9%)	.5815
Female	60	32	[22/10]	(54.2%)	28	(49.1%)	
Body mass index							
<18.5	18	11	[8/3]	(18.6%)	7	(12.3%)	.6326
18.5‐25	74	36	[19/17]	(61.0%)	38	(66.7%)	
>25	24	12	[8/4]	(20.3%)	12	(21.1%)	
CEA							
≤3.4	47	21	[13/8]	(35.6%)	26	(45.6%)	.2714
>3.4	69	38	[22/16]	(64.4%)	31	(54.4%)	
CA19‐9							
≤37	92	44	[24/20]	(74.6%)	48	(84.2%)	.1982
>37	24	15	[11/4]	(25.4%)	9	(15.8%)	
Stage							
I	10	6	[3/3]	(10.2%)	4	(7.0%)	.5055
II	40	18	[5/13 ]	(30.5%)	22	(38.6%)	
III	66	35	[25/10]	(59.3%)	31	(54.4%)	
ASA performance status							
1‐2	86	42	[23/19]	(71.2%)	44	(77.2%)	.5107
3‐	19	11	[8/3]	(18.6%)	8	(14.0%)	
Unknown	11	6	[2/4]	(59.3%)	5	(8.8%)	
Adjuvant chemotherapy							
None	64	34	[16/18]	(57.6%)	30	(52.6%)	.5899
Monotherapy	24	12	[8/4]	(20.3%)	12	(21.1%)	
Doublet therapy	20	8	[6/2]	(13.6%)	12	(21.1%)	
Unknown	8	5	[3/2]	(8.5%)	3	(5.3%)	
Palliative chemotherapy							
None	32	19	[9/10]	(32.2%)	13	(22.8%)	0.2076
Done	53	21	[13/8]	(35.6%)	32	(56.1%)	
Unknown	31	19	[11/8]	(32.2%)	12	(21.1%)	

Abbreviations: ASA, American Society of Anesthetists; CA19‐9, carbohydrate antigen 19‐9; CEA, carcinoembryonic antigen; L, left‐sided colon; R, right‐sided colon.

^a^ Significant difference.

**TABLE 2:** Number of recurrence organ sites in accordance with primary lesion

Number of recurrence organ sites	Total N = 116		Colon N = 59	[R/L]		Rectum N = 57		*P*‐value
One	91	(78.4%)	44	[24/20]	(74.6%)	47	(82.5%)	
Two	20	(17.2%)	12	[8/4]	(20.3%)	8	(14.0%)	.5401
Three or more	5	(4.3%)	3	[3/0]	(5.1%)	2	(3.5%)	

Abbreviations: L, left‐sided colon; R, right‐sided colon.

**TABLE 3:** Association between primary lesion and recurrence organ site

Recurrence organ	Total N = 91	Colon N = 44	[R/L]		Rectum N = 47		*P*‐value
Lung							
Present	32	13	[7/6]	(29.5%)	19	(40.4%)	.2763
Absent	59	31	[17/14]	(70.5%)	28	(59.6%)	
Liver							
Present	22	15	[8/7]	(34.1%)	7	(14.9%)	.0313^a^
Absent	69	29	[16/13]	(65.9%)	40	(85.1%)	
Local recurrence							
Present	14	3	[0/3]	(6.8%)	11	(23.4%)	.0408^a^, ^b^
Absent	77	41	[24/17]	(93.2%)	36	(76.6%)	
Lymph node							
Present	12	6	[4/2]	(13.6%)	6	(12.8%)	.9024
Absent	79	38	[20/18]	(86.4%)	41	(87.2%)	
Dissemination							
Present	9	7	[5/2]	(15.9%)	2	(4.3%)	.0838^b^
Absent	82	37	[19/18]	(84.1%)	45	(95.7%)	
Other organ							
Present	2	0	[0/0]	(0.0%)	2	(4.3%)	.4950^b^
Absent	89	44	[24/20]	(100.0%)	45	(95.7%)	

Abbreviations: L, left‐sided colon; R, right‐sided colon.

^a^ Statistically significant.

^b^ Fisher's exact test.

### 3.4 Evaluation of the association between recurrence organ site and survival

**FIGURE 2:**
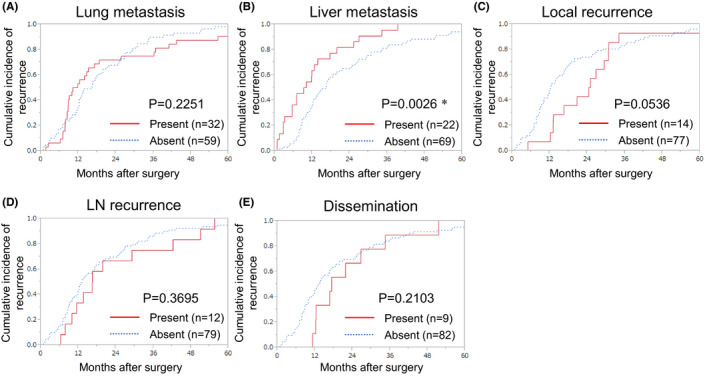
Recurrence rate according to the first recurrence organ (*P* value was estimated using the Wilcoxon signed‐rank test). Lung metastasis (A), liver metastasis (B), local recurrence (C), LN recurrence (D), and dissemination (E). LN, lymph node. *Statistically significant

**FIGURE 3:**
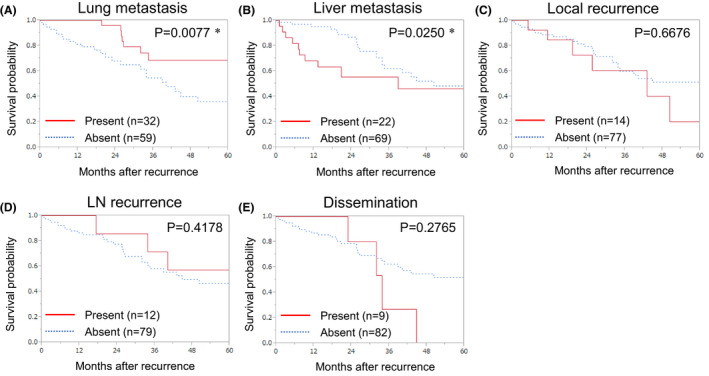
Overall survival after recurrence according to the first recurrence organ (*P* value was estimated using the log‐rank test). Lung metastasis (A), liver metastasis (B), local recurrence (C), LN recurrence (D), and dissemination (E). LN, lymph node. *Statistically significant

Liver, as the first recurrence site, was associated with an earlier recurrence after surgery as compared to other organ recurrences (*P* = .0026); local recurrence was detected later than other organ recurrences (*P* = .0536; Figure 2). Compared to the absence of liver metastasis, the presence of liver metastasis as the first recurrence was associated with a shorter OS after recurrence (HR: 2.216; 95% CI: 1.052–4.459; *P* = .0370). Compared to the absence of lung metastasis, the presence of lung metastasis as the first recurrence was associated with a longer OS after recurrence (HR: 0.338; 95% CI: 0.135–0.741; *P* = .0057; Figure 3). Of the 17 disseminations noted, eight (47.1%) were accompanied by other organ recurrences, and recurrences with accompanying dissemination were associated with a shorter OS after recurrence as compared to those without dissemination (HR: 2.962; 95% CI: 1.381–5.805; *P* = .0069; Figure S2).

### 3.5 Prognosis according to the recurrence patterns, invasive treatments, and adjuvant chemotherapy after primary resection

**FIGURE 4:**
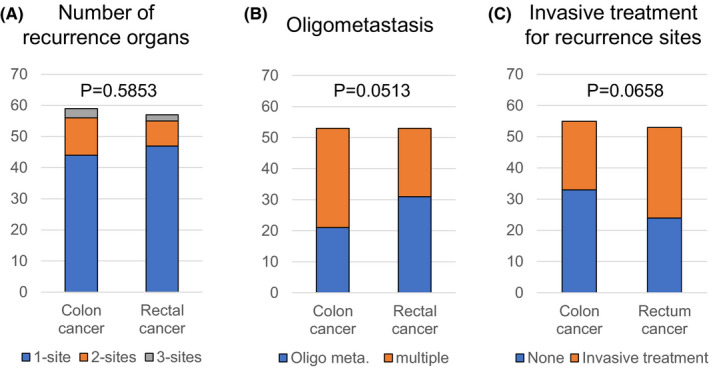
The number of patients according to the primary lesion. The number of recurrence organs (n = 116) (A), oligometastasis (n = 106; data on the number of lesions in a recurrent organ were unavailable for 10 patients with one‐organ recurrence) (B), and invasive treatment for recurrence sites (n = 108; data on invasive treatment were unavailable for eight patients) (C). Meta., metastasis

**FIGURE 5:**
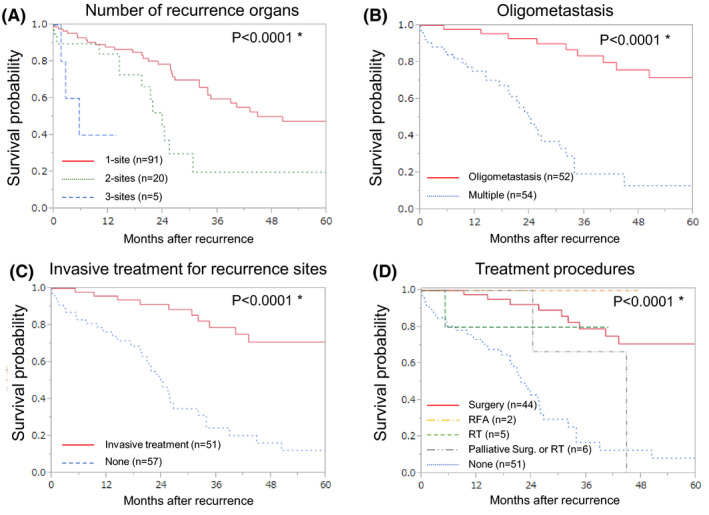
Kaplan–Meier survival curve according to the number of recurrence organs (n = 116) (A), oligometastasis (n = 106; data on the number of lesions in a recurrent organ were unavailable for 10 patients with one‐organ recurrence) (B), invasive treatment for recurrence sites (n = 108; data on invasive treatment were unavailable for eight patients) (C), and treatment procedures (n = 108) (D). *Statistically significant

The difference in the number of recurrence organ sites between right‐ or left‐sided CC and RC was not determined (*P* = .5853), but oligometastasis was observed more frequently in cases of RC than in those of right‐or left‐sided CC (*P* = .0513; Figure 4A,B). Patients with recurrence in multiple‐organ sites were associated with a shorter OS after recurrence as compared to those with recurrence in one‐organ sites (HR: 3.084; 95% CI: 1.597–5.707; *P* = .0012). Further, compared to those with oligometastasis, patients with multiple‐organ site metastasis were also associated with a shorter OS after recurrence (HR: 7.373; 95% CI: 3.608–16.360; *P* < .0001). The 5‐y OS rates of the patients with one recurrent organ site and oligometastasis were 47.5% and 71.7%, respectively (Figure 5A,B).

Invasive treatments tended to be performed more frequently for metastatic lesions in RC than for those in right‐or left‐sided CC (*P* = .0658), (Figure 4C). Patients who underwent invasive treatment for metastatic lesions had a longer OS after recurrence as compared to those who did not (HR: 0.184; 95% CI: 0.090–0.353; *P* < .0001; Figure 5C). A significant relationship between surgical procedures and prognosis was shown (*P* <.0001; Figure 5D).

The 5‐y OS after recurrence in patients who underwent invasive treatment was 71.9%. The median survival time in patients who underwent chemotherapy without invasive treatment was 24.3 mo and in patients who underwent neither invasive treatment nor chemotherapy was 8.6 mo (Figure S3).

**FIGURE 6:**
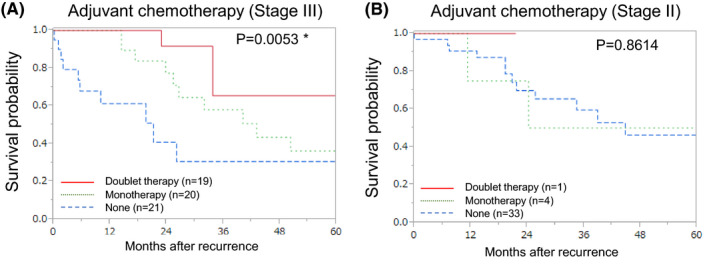
Kaplan–Meier survival curve according to adjuvant chemotherapy. Adjuvant chemotherapy for stage III patients (n = 60; data on adjuvant chemotherapy were unavailable for six patients) (A), adjuvant chemotherapy for Stage II patients (B) (n = 38; data on adjuvant chemotherapy were unavailable for two patients). *Statistically significant

The relationship between survival after recurrence and adjuvant chemotherapy after primary lesion resection was analyzed. Stage III patients who received adjuvant chemotherapy had a longer OS after recurrence as compared to stage III patients who did not receive adjuvant chemotherapy (*P* = .0053; Figure 6A). Survival after recurrence in Stage II patients did not differ with respect to adjuvant chemotherapy administration (Figure 6B).

### 3.6 Invasive treatments according to the recurrence organ

Invasive treatments for recurrence sites and the recurrence patterns after invasive treatment were evaluated according to the recurrence organ. Of the 14 patients with local recurrence, nine (64.3%) underwent an initial invasive treatment; one of these underwent a secondary invasive treatment for the lungs. Of the 32 patients with lung recurrence, 20 (62.5%) underwent invasive treatment and 10 (31.3%) underwent secondary treatment for re‐recurrence. Of these 10 patients, nine were treated for lung metastasis. Lung recurrence can be managed using several invasive treatments (Table 4), but recurrence accompanied by dissemination is difficult to treat, and only one patient underwent invasive treatment for this in our study (Table S5).

**TABLE 4:** Invasive treatment for recurrence in accordance with recurrence organ site

Rec. Organ	Total	Initial treatment	Secondary treatment							Third treatment						
	N	N	N	Liver	Lung	Local	LN	Diss.	Other	N	Liver	Lung	Local	LN	Diss.	Other
Lung	32	20 (62.5%)	10 (31.3%)	0	9	1	0	0	0	2 (6.3%)	0	1	1	0	0	0
Liver	22	11 (50.0%)	5 (22.7%)	3	2	0	0	0	0	1 (4.5%)	0	0	1	0	0	0
Local	14	9 (64.3%)	1 (7.1%)	0	1	0	0	0	0	1 (7.1%)	0	0	1	0	0	0
LN	12	5 (41.7%)	2 (16.7%)	1	0	0	1	0	0	0 (0.0%)	0	0	0	0	0	0
Diss.	9	1 (11.1%)	1 (11.1%)	0	1	0	0	0	0	0 (0.0%)	0	0	0	0	0	0
Site ≥ 2	25	3 (12.0%)	0 (0.0%)	0	0	0	0	0	0	0 (0.0%)	0	0	0	0	0	0

Note

Two patients were other site recurrence.

Abbreviations: diss., dissemination; LN, lymph node.

## 4 DISCUSSION

In this study the OS after recurrence in stage I–III CRC was evaluated with respect to the recurrence sites and patterns. Patients with recurrent RC had a longer OS after recurrence as compared to those with recurrent CC. The first recurrence of CC was more frequent in the liver as compared to that of RC and was accompanied by dissemination. However, liver metastasis and dissemination were associated with a poor prognosis after recurrence, and hence, treatment for recurrent lesions may focus on controlling tumor progression in the organs. On appropriate follow‐up, 50% of the recurrences were found to be oligometastases, which had favorable prognoses. Hence, for patients with oligometastasis, it is better to perform an aggressive treatment when possible.

The prognosis after recurrence of CRC was reported with respect to the primary lesions, stage at the primary surgery, and organ recurrence. O'Connell et al^9^ reported that the OS after recurrence was shorter in stage III CRC than in stage II CRC and that early recurrence was associated with a more unfavorable prognosis as compared to late recurrence. With respect to the primary lesion, the interval until recurrence was longer in cases of RC (26.0 ± 24.2 mo) as compared to in cases of CC (17.1 ± 11.0 mo; *P* = .03).^10^ Pugh et al^3^ also reported the prognosis after recurrence with respect to the stage of primary lesion, site of primary lesion, and site of recurrence; they found that an initial stage (*P* = .005) and the site of primary lesion influenced post‐recurrence survival. The results of our study were similar to those of previous studies with respect to the prognosis after recurrence and the primary location; however, the association between OS after recurrence and the first recurrence organ observed in our study offers a new insight. Local recurrence and lung metastasis are usually associated with RC. These recurrence patterns are likely due to the invasive and secondary treatments administered for re‐recurrence. Invasive treatments for local recurrence and lung metastasis may lead to a favorable prognosis for recurrent RC.

The prognosis after treatment of a recurrent organ, with respect to a metastatic lesion, has been reported previously. In previous meta‐analyses, the 5‐y OS rate following liver resection was approximately 38% (median survival: 3.6 y)^11^; it was 27%–68% after complete resection of lung metastasis^12^ and 36.4% after R0 resection for metachronous peritoneal metastasis.^13^ The risk factors associated with organ recurrence were also reported in these articles; however, the prognosis in relation to the first recurrence organ was not clear. We demonstrated that the OS after recurrence differed with the first organ recurrence. The prognosis of lung metastasis recurrence was favorable, and that of liver metastasis recurrence and accompanying dissemination was unfavorable. These data indicate that liver metastasis and dissemination may require the development of a more effective novel treatment strategy. Twelve patients experienced distant LN recurrence, 10 and 8 of which underwent D3 LN dissection and adjuvant chemotherapy, respectively. Biomarkers for predicting distant LN recurrence and a novel treatment strategy may be required for reducing LN recurrence. In a previous study, out of 119 recurrences, 93 (78%) involved a single organ and 26 (22%) involved multiple organs.^14^ These data were also similar to the data in our study with respect to the number of first recurrence organs. The interval from surgery to recurrence may be associated with first organ recurrence. In our study, liver metastasis occurred at an earlier stage as compared to other metastases. Roth et al^15^ reported that none of the patients had bone metastasis as the first metastatic lesion. Metastasis of CRC may occur in a limited area of an organ and then progress to various other organs.

We also evaluated the number of recurrences and the status of oligometastasis. Oligometastasis was a state of limited metastatic lesions. Treatment for all known metastatic lesions may lead to a cure, and further distant progression could be avoided.^16^ The impact of invasive treatment for oligometastasis was reported in several randomized controlled trails (RCTs). In one RCT, compared to conventional treatment, stereotactic ablative radiotherapy (SABR) improved the prognosis for solid tumors (median OS [control group vs. SABR group]: 28 mo [95% CI: 19–33 mo] vs. 41 mo [95% CI: 26―not reached]; hazard ratio: 0.57, 95% CI: 0.30–1.10; *P* = .090).^17^ The inclusion criteria in this RCT were the presence of controlled primary tumors and up to five metastases. In our study, the primary lesion was resected and we defined both one‐organ recurrence and the presence of fewer than five lesions in the organ as oligometastasis. It has been reported that metastasis‐directed therapy also improves prognosis of oligometastasis in prostate cancer.^18^ In that study, the number of patients with one recurrence organ site and oligometastasis was 91/116 (78.4%) and 52/106 (49.1%), respectively. Half of the patients who experienced recurrence were diagnosed with oligometastasis based on the follow‐up guidelines in the study. Furthermore, the 5‐y OS rates of the patients with one recurrence organ site and oligometastasis were 47.5% and 71.7%, respectively. The survival curve of patients with oligometastasis was closer to the survival curve of patients with stage III CRC than to the curve of those with stage IV CRC. These data support the use of aggressive treatments in patients with oligometastasis when possible.

This study has some limitations. First, it was a retrospective study, and the treatment strategy employed for the recurrence site was at the personal discretion of our medical team. Second, although we noted treatments performed in accordance with the treatment guidelines, the treatment outcomes differed between the institutions.

In conclusion, we found that with respect to the first recurrence site, liver metastasis was associated with an earlier recurrence in patients with stage I–III CRC. Patients with recurrence in the liver and accompanying dissemination had a shorter OS after recurrence as compared to those with other organ recurrences. Patients with recurrences in the lung had a more favorable prognosis as compared to those with other organ recurrences. The number of metastatic organs and invasive treatments were associated with patient prognosis. Future investigation into treatment strategies with respect to the number and organ of first recurrence may be useful in improving the prognosis of CRC patients after recurrence.

## DISCLOSURE

Funding: No funding was received for this study.

Conflict of Interest: The authors declare no conflicts of interest for this article.

## Supporting information

Figure S1

Figure S2

Figure S3

Table S1

Table S2

Table S3

Table S4

Table S5

## References

[1] Torre LA , Bray F , Siegel RL , Ferlay J , Lortet‐Tieulent J , Jemal A . Global cancer statistics, 2012. CA Cancer J Clin. 2015;65(2):87–108. 25651787 10.3322/caac.21262

[2] Hashiguchi Y , Muro K , Saito Y , Ito Y , Ajioka Y , Hamaguchi T , et al. Japanese Society for Cancer of the Colon and Rectum (JSCCR) guidelines 2019 for the treatment of colorectal cancer. Int J Clin Oncol. 2020;25(1):1–42. 31203527 10.1007/s10147-019-01485-z PMC6946738

[3] Pugh SA , Shinkins B , Fuller A , Mellor J , Mant D , Primrose JN . Site and stage of colorectal cancer influence the likelihood and distribution of disease recurrence and postrecurrence survival: data from the FACS randomized controlled trial. Ann Surg. 2016;263(6):1143–7. 26135689 10.1097/SLA.0000000000001351

[4] Hellman S , Weichselbaum RR . Oligometastases. J Clin Oncol. 1995;13(1):8–10. 7799047 10.1200/JCO.1995.13.1.8

[5] Poon I , Erler D , Dagan R , Redmond KJ , Foote M , Badellino S , et al. Evaluation of definitive stereotactic body radiotherapy and outcomes in adults with extracranial oligometastasis. JAMA Netw Open. 2020;3(11):e2026312. 33196810 10.1001/jamanetworkopen.2020.26312 PMC7670310

[6] Takeda A , Sanuki N , Kunieda E . Role of stereotactic body radiotherapy for oligometastasis from colorectal cancer. World J Gastroenterol. 2014;20(15):4220–9. 24764660 10.3748/wjg.v20.i15.4220 PMC3989958

[7] Holch JW , Ricard I , Stintzing S , Modest DP , Heinemann V . The relevance of primary tumour location in patients with metastatic colorectal cancer: a meta‐analysis of first‐line clinical trials. Eur J Cancer. 2017;70:87–98. 27907852 10.1016/j.ejca.2016.10.007

[8] Japanese classification of colorectal, appendiceal, and anal carcinoma: the 3d English Edition [Secondary Publication]. J Anus Rectum Colon. 2019;3(4):175–95. 31768468 10.23922/jarc.2019-018 PMC6845287

[9] O'Connell MJ , Campbell ME , Goldberg RM , Grothey A , Seitz JF , Benedetti JK , et al. Survival following recurrence in stage II and III colon cancer: findings from the ACCENT data set. J Clin Oncol. 2008;26(14):2336–41. 18467725 10.1200/JCO.2007.15.8261

[10] Sadahiro S , Suzuki T , Ishikawa K , Nakamura T , Tanaka Y , Masuda T , et al. Recurrence patterns after curative resection of colorectal cancer in patients followed for a minimum of ten y. Hepatogastroenterology. 2003;50(53):1362–6. 14571738

[11] Kanas GP , Taylor A , Primrose JN , Langeberg WJ , Kelsh MA , Mowat FS , et al. Survival after liver resection in metastatic colorectal cancer: review and meta‐analysis of prognostic factors. Clin Epidemiol. 2012;4:283–301. 23152705 10.2147/CLEP.S34285 PMC3496330

[12] Gonzalez M , Poncet A , Combescure C , Robert J , Ris HB , Gervaz P . Risk factors for survival after lung metastasectomy in colorectal cancer patients: a systematic review and meta‐analysis. Ann Surg Oncol. 2013;20(2):572–9. 23104709 10.1245/s10434-012-2726-3

[13] Sato H , Maeda K , Kotake K , Sugihara K , Takahashi H . Factors affecting recurrence and prognosis after R0 resection for colorectal cancer with peritoneal metastasis. J Gastroenterol. 2016;51(5):465–72. 26377391 10.1007/s00535-015-1122-8

[14] Ding P , Liska D , Tang P , Shia J , Saltz L , Goodman K , et al. Pulmonary recurrence predominates after combined modality therapy for rectal cancer: an original retrospective study. Ann Surg. 2012;256(1):111–6. 22664562 10.1097/SLA.0b013e31825b3a2b

[15] Roth ES , Fetzer DT , Barron BJ , Joseph UA , Gayed IW , Wan DQ . Does colon cancer ever metastasize to bone first? a temporal analysis of colorectal cancer progression. BMC Cancer. 2009;9:274. 19664211 10.1186/1471-2407-9-274 PMC2734866

[16] Palma DA , Louie AV , Rodrigues GB . New strategies in stereotactic radiotherapy for oligometastases. Clin Cancer Res. 2015;21(23):5198–204. 26626571 10.1158/1078-0432.CCR-15-0822

[17] Palma DA , Olson R , Harrow S , Gaede S , Louie AV , Haasbeek C , et al. Stereotactic ablative radiotherapy versus standard of care palliative treatment in patients with oligometastatic cancers (SABR‐COMET): a randomised, phase 2, open‐label trial. Lancet. 2019;393(10185):2051–8. 30982687 10.1016/S0140-6736(18)32487-5

[18] Phillips R , Shi WY , Deek M , Radwan N , Lim SJ , Antonarakis ES , et al. Outcomes of observation vs. stereotactic ablative radiation for oligometastatic prostate cancer: the ORIOLE phase 2 randomized clinical trial. JAMA Oncol. 2020;6(5):650–9. 32215577 10.1001/jamaoncol.2020.0147 PMC7225913

