# Supplementary material for: Overall survival after recurrence in stage I–III colorectal cancer patients in accordance with the recurrence organ site and pattern
Source: Ann Gastroenterol Surg. 2021 Jul 14;5(6):813–22. doi: 10.1002/ags3.12483 (PMC8560596; doi:10.1002/ags3.12483)
Supplement: Supplementary file 1 — Figure S1 [file AGS3-5-813-s002.pptx]

## Slide 1
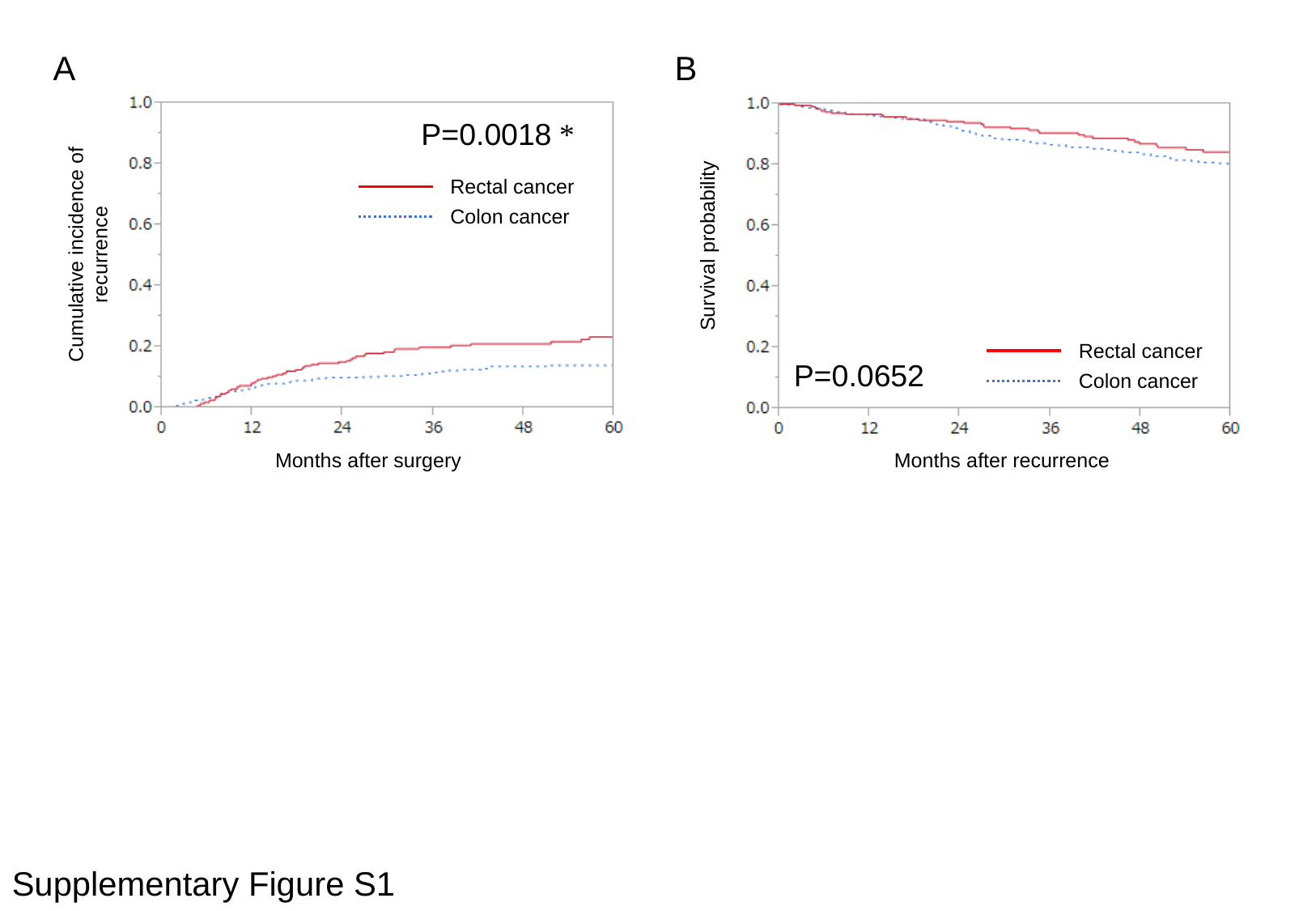

A
B
P=0.0018 *
Rectal cancer
Colon cancer
Survival probability
Cumulative incidence of recurrence
Rectal cancer
P=0.0652
Colon cancer
Months after surgery
Months after surgery
Months after recurrence
Supplementary Figure S1
