# Supplementary material for: Overall survival after recurrence in stage I–III colorectal cancer patients in accordance with the recurrence organ site and pattern
Source: Ann Gastroenterol Surg. 2021 Jul 14;5(6):813–22. doi: 10.1002/ags3.12483 (PMC8560596; doi:10.1002/ags3.12483)
Supplement: Supplementary file 2 — Figure S2 [file AGS3-5-813-s003.pptx]

## Slide 1
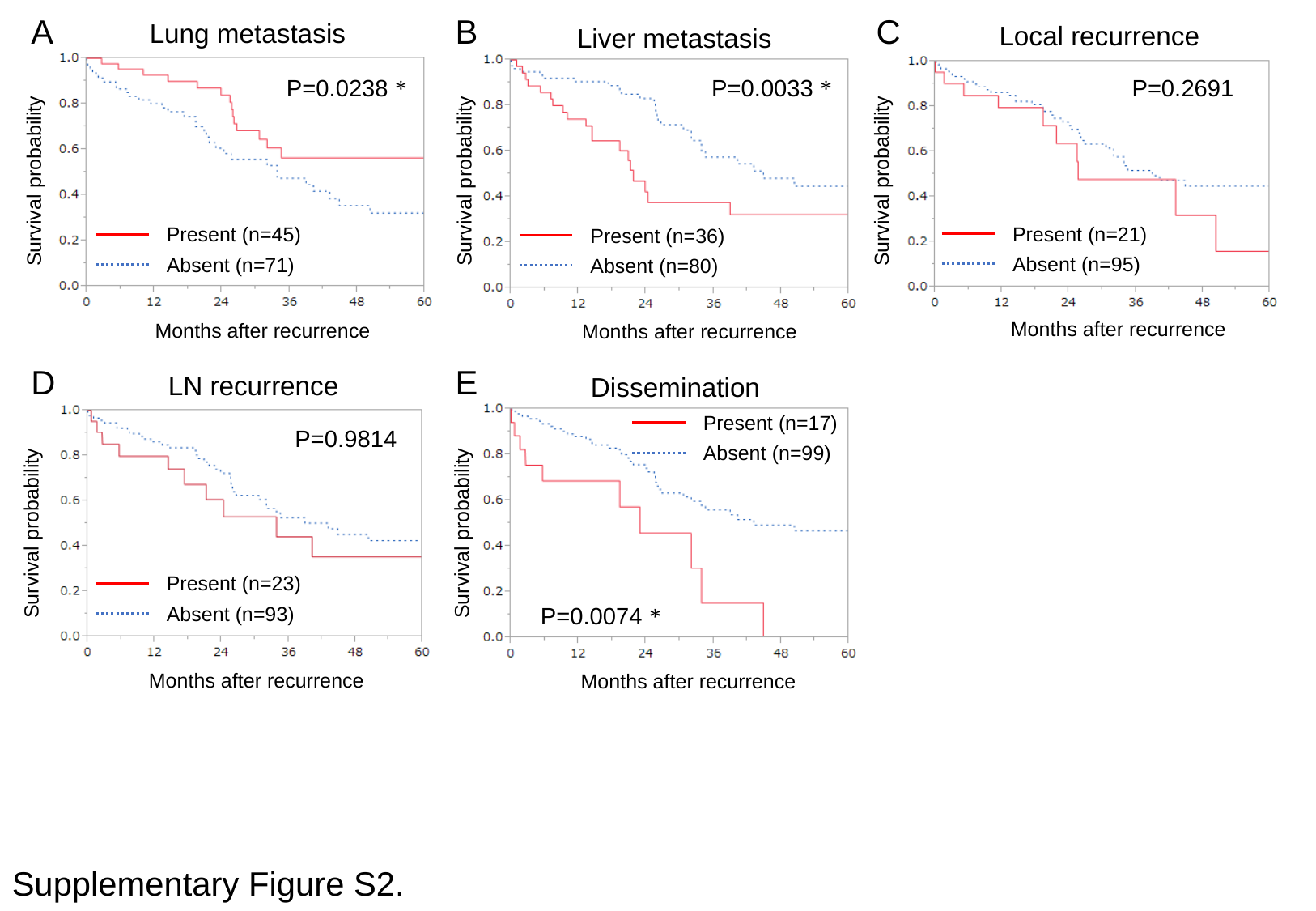

A
B
C
Lung metastasis
Local recurrence
Liver metastasis
P=0.0033 *
P=0.2691
P=0.0238 *
Survival probability
Survival probability
Survival probability
Present (n=21)
Present (n=45)
Present (n=36)
Absent (n=95)
Absent (n=71)
Absent (n=80)
Months after recurrence
Months after recurrence
Months after recurrence
D
E
LN recurrence
Dissemination
Present (n=17)
P=0.9814
Absent (n=99)
Survival probability
Survival probability
Present (n=23)
P=0.0074 *
Absent (n=93)
Months after recurrence
Months after recurrence
Supplementary Figure S2.
