# Supplementary material for: Overall survival after recurrence in stage I–III colorectal cancer patients in accordance with the recurrence organ site and pattern
Source: Ann Gastroenterol Surg. 2021 Jul 14;5(6):813–22. doi: 10.1002/ags3.12483 (PMC8560596; doi:10.1002/ags3.12483)
Supplement: Supplementary file 3 — Figure S3 [file AGS3-5-813-s004.pptx]

## Slide 1
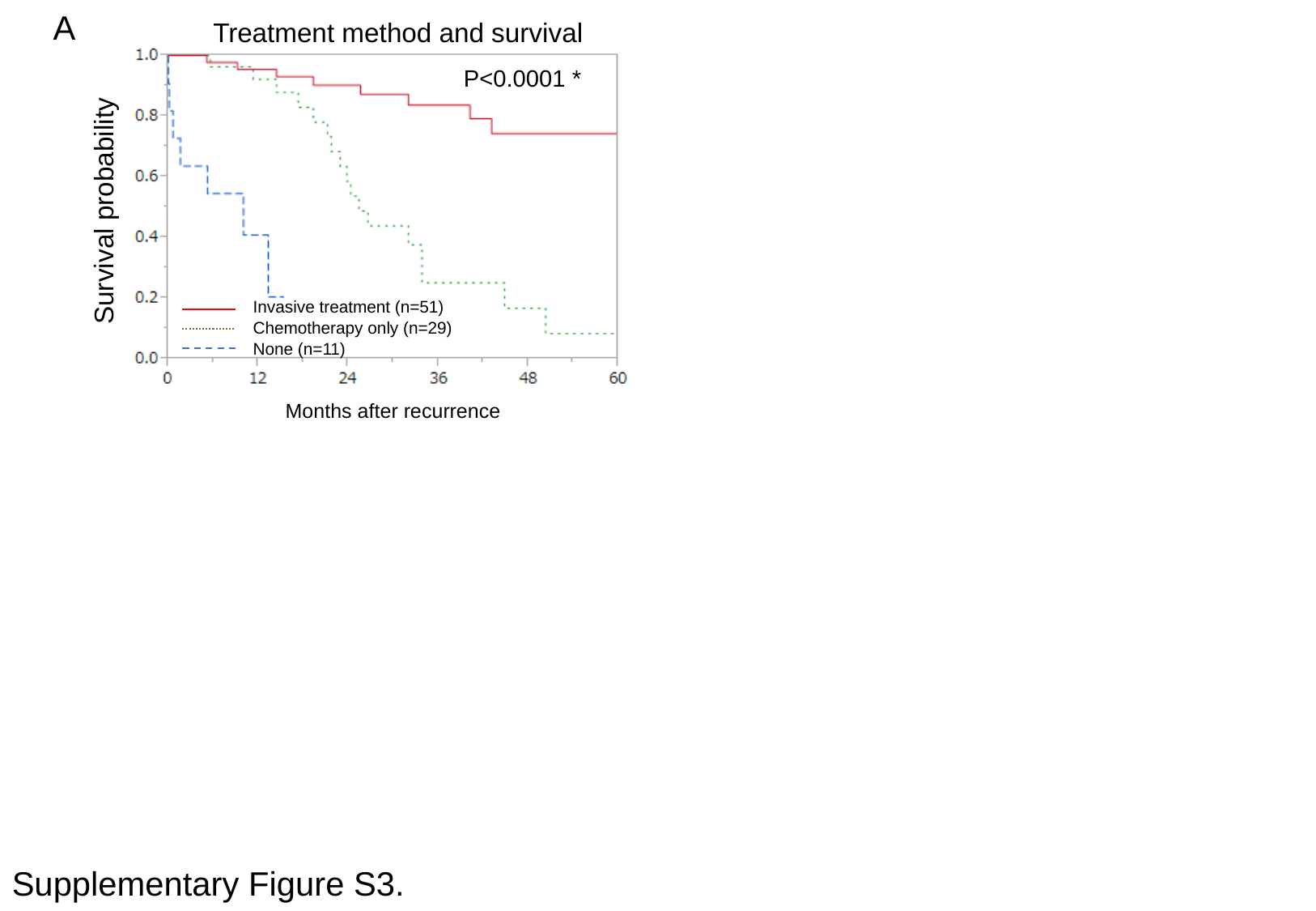

A
Treatment method and survival
P<0.0001 *
Survival probability
Invasive treatment (n=51)
Chemotherapy only (n=29)
None (n=11)
Months after recurrence
Supplementary Figure S3.
