# Supplementary material for: Overall survival after recurrence in stage I–III colorectal cancer patients in accordance with the recurrence organ site and pattern
Source: Ann Gastroenterol Surg. 2021 Jul 14;5(6):813–22. doi: 10.1002/ags3.12483 (PMC8560596; doi:10.1002/ags3.12483)
Supplement: Supplementary file 4 — Table S1 [file AGS3-5-813-s008.docx]

**Supplementary Table S1.** **Association between the primary lesion and clinicopathological factors.**

| Factor | Total N | Colon N=488 | [ | R | / | L | ] |  |  |  |  |  | Rectum N=275 |  |  |  |  | *P-value* |  |
| --- | --- | --- | --- | --- | --- | --- | --- | --- | --- | --- | --- | --- | --- | --- | --- | --- | --- | --- | --- |
| Age |  |  |  |  |  |  |  |  |  |  |  |  |  |  |  |  |  | <0.0001 * |  |
| ≤ 70 | 417 | 229 | [ | 101 | / | 128 | ] | ( | 46.9 | % | ) |  | 188 | ( | 68.4 | % | ) |  |  |
| > 70 | 346 | 259 | [ | 146 | / | 113 | ] | ( | 53.1 | % | ) |  | 87 | ( | 31.6 | % | ) |  |  |
| Gender |  |  |  |  |  |  |  |  |  |  |  |  |  |  |  |  |  | 0.6156 |  |
| Male | 432 | 273 | [ | 133 | / | 140 | ] | ( | 55.9 | % | ) |  | 159 | ( | 57.8 | % | ) |  |  |
| Female | 331 | 215 | [ | 114 | / | 101 | ] | ( | 44.1 | % | ) |  | 116 | ( | 42.2 | % | ) |  |  |
| Body mass index |  |  |  |  |  |  |  |  |  |  |  |  |  |  |  |  |  | 0.4934 |  |
| < 18.5 | 88 | 52 | [ | 32 | / | 20 | ] | ( | 10.7 | % | ) |  | 36 | ( | 13.1 | % | ) |  |  |
| 18.5-25 | 504 | 329 | [ | 160 | / | 169 | ] | ( | 67.4 | % | ) |  | 175 | ( | 63.6 | % | ) |  |  |
| > 25 | 171 | 107 | [ | 55 | / | 52 | ] | ( | 21.9 | % | ) |  | 64 | ( | 23.3 | % | ) |  |  |
| CEA |  |  |  |  |  |  |  |  |  |  |  |  |  |  |  |  |  | 0.7381 |  |
| ≤ 3.4 | 436 | 281 | [ | 137 | / | 144 | ] | ( | 57.6 | % | ) |  | 155 | ( | 56.4 | % | ) |  |  |
| > 3.4 | 324 | 205 | [ | 109 | / | 96 | ] | ( | 42.0 | % | ) |  | 119 | ( | 43.3 | % | ) |  |  |
| CA19-9 |  |  |  |  |  |  |  |  |  |  |  |  |  |  |  |  |  | 0.8997 |  |
| ≤ 37 | 665 | 425 | [ | 214 | / | 211 | ] | ( | 87.1 | % | ) |  | 240 | ( | 87.3 | % | ) |  |  |
| > 37 | 87 | 55 | [ | 30 | / | 25 | ] | ( | 11.3 | % | ) |  | 32 | ( | 11.6 | % | ) |  |  |
| Stage |  |  |  |  |  |  |  |  |  |  |  |  |  |  |  |  |  | 0.0031 * |  |
| I | 267 | 181 | [ | 88 | / | 93 | ] | ( | 37.1 | % | ) |  | 86 | ( | 31.3 | % | ) |  |  |
| II | 268 | 182 | [ | 79 | / | 103 | ] | ( | 37.3 | % | ) |  | 86 | ( | 31.3 | % | ) |  |  |
| III | 228 | 125 | [ | 80 | / | 45 | ] | ( | 25.6 | % | ) |  | 103 | ( | 37.5 | % | ) |  |  |
| Depth of invasion |  |  |  |  |  |  |  |  |  |  |  |  |  |  |  |  |  | 0.5883 |  |
| pT1b-T2 | 315 | 205 | [ | 101 | / | 104 | ] | ( | 42.0 | % | ) |  | 110 | ( | 40.0 | % | ) |  |  |
| pT3-T4 | 448 | 283 | [ | 146 | / | 137 | ] | ( | 58.0 | % | ) |  | 165 | ( | 60.0 | % | ) |  |  |
| Lymph node metastasis |  |  |  |  |  |  |  |  |  |  |  |  |  |  |  |  |  | 0.0007 * |  |
| Absent | 535 | 363 | [ | 167 | / | 196 | ] | ( | 74.4 | % | ) |  | 172 | ( | 62.5 | % | ) |  |  |
| Present | 228 | 125 | [ | 80 | / | 45 | ] | ( | 25.6 | % | ) |  | 103 | ( | 37.5 | % | ) |  |  |
| Lymphatic invasion |  |  |  |  |  |  |  |  |  |  |  |  |  |  |  |  |  | 0.1398 |  |
| Absent | 618 | 403 | [ | 191 | / | 212 | ] | ( | 82.6 | % | ) |  | 215 | ( | 78.2 | % | ) |  |  |
| Present | 145 | 85 | [ | 56 | / | 29 | ] | ( | 17.4 | % | ) |  | 60 | ( | 21.8 | % | ) |  |  |
| Vascular invasion |  |  |  |  |  |  |  |  |  |  |  |  |  |  |  |  |  | 0.0573 |  |
| Absent | 379 | 255 | [ | 118 | / | 137 | ] | ( | 52.3 | % | ) |  | 124 | ( | 45.1 | % | ) |  |  |
| Present | 384 | 233 | [ | 129 | / | 104 | ] | ( | 47.7 | % | ) |  | 151 | ( | 54.9 | % | ) |  |  |

R: right-sided colon, L: left-sided colon, CEA: carcinoembryonic antigen, CA19-9: carbohydrate antigen 19-9, * Significant difference
