# Supplementary material for: Overall survival after recurrence in stage I–III colorectal cancer patients in accordance with the recurrence organ site and pattern
Source: Ann Gastroenterol Surg. 2021 Jul 14;5(6):813–22. doi: 10.1002/ags3.12483 (PMC8560596; doi:10.1002/ags3.12483)
Supplement: Supplementary file 5 — Table S2 [file AGS3-5-813-s005.docx]

**Supplementary Table S2. Analysis of recurrence in patients with CRC using univariate and multivariate Cox proportional hazards analysis.**

|  |  |  | Univariate analysis | | |  | Multivariate analysis | | |  |
| --- | --- | --- | --- | --- | --- | --- | --- | --- | --- | --- |
|  | Factors | Ref. | HR | 95% CI | P-value |  | HR | 95% CI | P-value |  |
|  | Age: ≥ 70 | < 70 | 1.167 | (0.807-1.680) | 0.409 |  | 1.245 | (0.852-1.812) | 0.254 |  |
|  | Gender: Male | Female | 0.669 | (0.464-0.964) | 0.031 * |  | 0.789 | (0.539-1.155) | 0.222 |  |
|  | CEA: >3.4 | ≤ 3.4 | 2.411 | (1.667-3.521) | <0.001 * |  | 1.525 | (1.026-2.282) | 0.037 * |  |
|  | CA19-9: >37 | ≤ 37 | 2.312 | (1.443-3.561) | <0.001 * |  | 1.582 | (0.964-2.509) | 0.069 |  |
|  | Depth of invasion: pT3-4 | pT1-2 | 5.815 | (3.445-10.625) | <0.001 * |  | 3.895 | (2.246-7.264) | <0.001 * |  |
|  | LN metastasis: Present | Absent | 3.678 | (2.551-5.336) | <0.001 * |  | 2.460 | (1.683-3.622) | <0.001 * |  |
|  | Tumor location: Rectum | Colon | 1.774 | (1.231-2.555) | 0.002 * |  | 1.697 | (1.163-2.476) | 0.006 * |  |

HR: hazard ratio, CI: confidence interval, LN: lymph node, CEA: carcinoembryonic antigen, CA19-9: carbohydrate antigen 19-9, * Significant difference.
