# Supplementary material for: Overall survival after recurrence in stage I–III colorectal cancer patients in accordance with the recurrence organ site and pattern
Source: Ann Gastroenterol Surg. 2021 Jul 14;5(6):813–22. doi: 10.1002/ags3.12483 (PMC8560596; doi:10.1002/ags3.12483)
Supplement: Supplementary file 6 — Table S3 [file AGS3-5-813-s007.docx]

**Supplementary Table S3. Analysis of overall survival of CRC patients in univariate and multivariate Cox proportional hazards analysis.**

|  |  |  | Univariate analysis | | |  | Multivariate analysis | | |  |
| --- | --- | --- | --- | --- | --- | --- | --- | --- | --- | --- |
|  | Factors | Ref. | HR | 95% CI | P-value |  | HR | 95% CI | P-value |  |
|  | Age: ≥ 70 | < 70 | 2.661 | (1.864-3.851) | <0.001 * |  | 2.507 | (1.726-3.694) | <0.001 * |  |
|  | Gender: Male | Female | 1.115 | (0.785-1.599) | 0.545 |  | 1.278 | (0.877-1.883) | 0.203 |  |
|  | CEA: >3.4 | ≤ 3.4 | 1.940 | (1.371-2.756) | <0.001 * |  | 1.776 | (1.220-2.590) | 0.003 * |  |
|  | CA19-9: >37 | ≤ 37 | 1.495 | (0.899-2.362) | 0.117 |  | 1.227 | (0.718-2.002) | 0.442 |  |
|  | Depth of invasion: pT3-4 | pT1-2 | 1.497 | (1.045-2.176) | 0.028 * |  | 1.132 | (0.764-1.700) | 0.539 |  |
|  | LN metastasis: Present | Absent | 1.503 | (1.044-2.140) | 0.029 * |  | 1.422 | (0.963-2.079) | 0.076 |  |
|  | Tumor location: Rectum | Colon | 0.698 | (0.470-1.015) | 0.060 |  | 0.763 | (0.500-1.139) | 0.190 |  |

HR: hazard ratio, CI: confidence interval, LN: lymph node, CEA: carcinoembryonic antigen, CA19-9: carbohydrate antigen 19-9, * Significant difference.
