# Supplementary material for: Overall survival after recurrence in stage I–III colorectal cancer patients in accordance with the recurrence organ site and pattern
Source: Ann Gastroenterol Surg. 2021 Jul 14;5(6):813–22. doi: 10.1002/ags3.12483 (PMC8560596; doi:10.1002/ags3.12483)
Supplement: Supplementary file 7 — Table S4 [file AGS3-5-813-s006.docx]

**Supplementary Table S4. The association between primary lesion and accompanied recurrence organ site** **(in some patients, the first recurrence site was more than one site).**

| Accompanied recurrence organ sites | Total N=116 | Colon N=59 | [ | R | / | L | ] |  |  |  |  |  | Rectum N=57 |  |  |  |  | *P-value* |  |  |
| --- | --- | --- | --- | --- | --- | --- | --- | --- | --- | --- | --- | --- | --- | --- | --- | --- | --- | --- | --- | --- |
| Lung |  |  |  |  |  |  |  |  |  |  |  |  |  |  |  |  |  | 0.0618 |  |  |
| Present | 45 | 18 | [ | 11 | / | 7 | ] | ( | 30.5 | % | ) |  | 27 | ( | 47.4 | % | ) |  |  |  |
| Absent | 71 | 41 | [ | 24 | / | 17 | ] | ( | 69.5 | % | ) |  | 30 | ( | 52.6 | % | ) |  |  |  |
| Liver |  |  |  |  |  |  |  |  |  |  |  |  |  |  |  |  |  | 0.0277 | * |  |
| Present | 36 | 24 | [ | 14 | / | 10 | ] | ( | 40.7 | % | ) |  | 12 | ( | 21.1 | % | ) |  |  |  |
| Absent | 80 | 35 | [ | 21 | / | 14 | ] | ( | 59.3 | % | ) |  | 45 | ( | 78.9 | % | ) |  |  |  |
| Lymph node |  |  |  |  |  |  |  |  |  |  |  |  |  |  |  |  |  | 0.2819 |  |  |
| Present | 23 | 14 | [ | 10 | / | 4 | ] | ( | 23.7 | % | ) |  | 9 | ( | 15.8 | % | ) |  |  |  |
| Absent | 93 | 45 | [ | 25 | / | 20 | ] | ( | 76.3 | % | ) |  | 48 | ( | 84.2 | % | ) |  |  |  |
| Local recurrence |  |  |  |  |  |  |  |  |  |  |  |  |  |  |  |  |  | 0.0222 | * |  |
| Present | 21 | 6 | [ | 3 | / | 3 | ] | ( | 10.2 | % | ) |  | 15 | ( | 26.3 | % | ) |  |  |  |
| Absent | 95 | 53 | [ | 32 | / | 21 | ] | ( | 89.8 | % | ) |  | 42 | ( | 73.7 | % | ) |  |  |  |
| Dissemination |  |  |  |  |  |  |  |  |  |  |  |  |  |  |  |  |  | 0.0073 | * | § |
| Present | 17 | 14 | [ | 10 | / | 4 | ] | ( | 23.7 | % | ) |  | 3 | ( | 5.3 | % | ) |  |  |  |
| Absent | 99 | 45 | [ | 25 | / | 20 | ] | ( | 76.3 | % | ) |  | 54 | ( | 94.7 | % | ) |  |  |  |
| Other organ |  |  |  |  |  |  |  |  |  |  |  |  |  |  |  |  |  | 0.4346 |  | § |
| Present | 6 | 2 | [ | 2 | / | 0 | ] | ( | 3.4 | % | ) |  | 4 | ( | 7.0 | % | ) |  |  |  |
| Absent | 110 | 57 | [ | 33 | / | 24 | ] | ( | 96.6 | % | ) |  | 53 | ( | 93.0 | % | ) |  |  |  |

R: right-sided colon, L: left-sided colon, * statistical significant, § Fisher’s exact test
