# Supplementary material for: Overall survival after recurrence in stage I–III colorectal cancer patients in accordance with the recurrence organ site and pattern
Source: Ann Gastroenterol Surg. 2021 Jul 14;5(6):813–22. doi: 10.1002/ags3.12483 (PMC8560596; doi:10.1002/ags3.12483)
Supplement: Supplementary file 8 — Table S5 [file AGS3-5-813-s001.docx]

**Supplementary Table S5. Invasive treatment for recurrence in accordance with recurrence organ site** (**in some patients, the first recurrence site was more than one site).**

| Rec. Organ | Total | Initial treatment |  | Secondary treatment | | | | | | |  | Third treatment | | | | | | |
| --- | --- | --- | --- | --- | --- | --- | --- | --- | --- | --- | --- | --- | --- | --- | --- | --- | --- | --- |
|  | N | N |  | N | liver | lung | local | LN | diss. | other |  | N | Liver | lung | local | LN | diss. | other |
| lung | 45 | 21 (46.7%) |  | 11 (24.4%) | 0 | 10 | 1 | 0 | 0 | 0 |  | 2 (4.4%) | 0 | 1 | 1 | 0 | 0 | 0 |
| liver | 36 | 13 (36.1%) |  | 5 (13.9%) | 3 | 2 | 0 | 0 | 0 | 0 |  | 1 (2.8%) | 0 | 0 | 1 | 0 | 0 | 0 |
| local | 21 | 9 (42.9%) |  | 1 (4.8%) | 0 | 1 | 0 | 0 | 0 | 0 |  | 1 (4.8%) | 0 | 0 | 1 | 0 | 0 | 0 |
| LN | 23 | 7 (30.4%) |  | 2 (8.7%) | 1 | 0 | 0 | 1 | 0 | 0 |  | 0 (0.0%) | 0 | 0 | 0 | 0 | 0 | 0 |
| diss. | 17 | 1 (5.9%) |  | 0 (0.0%) | 0 | 0 | 0 | 0 | 0 | 0 |  | 0 (0.0%) | 0 | 0 | 0 | 0 | 0 | 0 |

LN: lymph node, diss.: dissemination
